# Exploring the longitudinal dynamics of self-criticism, self-compassion, psychological flexibility, and mental health in a three-wave study

**DOI:** 10.1038/s41598-025-95821-1

**Published:** 2025-04-22

**Authors:** Ming Yu Claudia Wong, Hong Wang Fung, Janet Yuen-Ha Wong, Stanley Kam Ki Lam

**Affiliations:** 1 Department of Health and Physical Education, The Education University of Hong Kong, Tai Po, Hong Kong; 2 School of Nursing, The Hong Kong Polytechnic University, Hung Hom, Hong Kong; 3 School of Nursing and Health Studies, Hong Kong Metropolitan University, Ho Man Tin, Hong Kong; 4 Nethersole School of Nursing, Faculty of Medicine, The Chinese University of Hong Kong, Ma Liu Shui, Hong Kong

**Keywords:** Self-criticism, Self-compassion, Mental health, Longitudinal, Psychology, Health care

## Abstract

Self-compassion has been emphasized in its association with reducing anxiety, narcissism, and self-criticism. At the same time, self-judgment as the counter side of self-kindness tends to interchange with the description of self-criticism, which can lead to potential stress and mental illness. Meanwhile, psychological flexibility enhanced through Acceptance and Commitment Therapy (ACT) emerges and is engaged as a therapeutic action after self-compassion. Hence, based on The Transactional Model of Stress and Coping, this three-wave longitudinal study examined longitudinal connections between (1) cognitive appraisal – self-criticism (comparative self-criticism and internalized self-criticism); (2) coping – self-compassion and psychological flexibility (acceptance and action), and (3) outcome – mental health. Regarding the results, at baseline, 412 participants (M age = 39.73, SD = 12.75; 83% female) were enrolled; follow-up rates were 56% at 6 months and 28%(N=115, M age = 40.88, SD = 13.00, 78.3% female) at 12 months. Using the Repeated Measures Panel Analysis Framework, the model investigation with the good fit model index supports the hypothesized pathways based on the Transactional Model of Stress and Coping. Self-compassion and psychological flexibility have been examined to be consistent and stable coping strategies negatively associated with self-criticism. Hence, the current study outcome serves as a theoretical foundation that supports the development of the intervention, evidenced by the potential mediating role or function of self-compassion or compassion-focused therapy and enhancing psychological flexibility through acceptance and commitment therapy.

## Introduction

Feelings of guilt, failure, and unworthiness are common in self-critical people. They constantly and harshly examine and assess themselves, fearing rejection and criticism and losing other people’s acceptance and approval. Self-criticism, accompanied by negative feelings like rage and self-contempt, is the process of negatively evaluating and scrutinizing oneself^1^. These characteristics lead to self-criticism as part of perfectionism or even leading to maladjustment^2^. Moreover, research has indicated self-criticism includes ‘hated self’ and the ‘inadequate self’, which represent distinct forms of self-criticizing characteristics and are associated with various psychological distress^3,4^. The ‘hated-self’ form of self-criticism involves aggression, self-hatred, and a self-deprecating attitude driven by a strong desire to eliminate perceived bad qualities inside oneself. It has also been mentioned in another self-criticism construct by^5^ indicating people with high levels of Internalized Self-Criticism (ISC) dedicated to high levels of internal standards and self-evaluation thus involving harsh self-judgment based on personal standards and expectations, leading to feelings of failure and worthlessness. Meanwhile, “inadequate self” highlights a person who is concerned about deficiencies, areas for improvement, and failures with the purpose of self-correction. Notwithstanding the other forms of self-criticism under^5^ construct – Comparative Self-Criticism (CSC) also involves comparing oneself unfavorably to others, leading to feelings of inferiority and inadequacy. The negative correlation between forms of self-criticism and self-esteem, life satisfaction, neuroticism, conflict resolution, and self-compassion among clinical and nonclinical populations has been observed^5–7^.

Additionally, self-critical individuals tend to be sensitive to disapproval or criticism from others and are achievement-oriented. They are also more likely to appraise achievement-related events negatively, demonstrate heightened ambivalence, and exhibit self-defeating behaviors^8^. Self-criticism is associated with more self-presentation goals and fewer interpersonal goals. A negative association has also been found between self-criticism and self-reported goal progress^9^; thus associating with increased negative psychological aspects^10^. Hence, it is crucial to take into account the many manifestations of self-criticism in relation to psychological suffering, which serves as a predisposing factor for depression and other mental health illnesses. Self-criticism is detrimental to both clinical disorders and interpersonal relationships, and being defined as coldness versus self-compassion as warmth, self-compassion can be considered a positive tool to reduce self-criticism, leading to cherishing emotional well-being^11^.

Self-compassion is a hearty way of developing a positive self-relationship, with the three basic elements overlapping and interacting with each other^12^. These dimensions include (1) treating oneself with kindness as opposed to harsh judgment or self-criticism (self-kindness versus self-judgment); (2) acknowledging that suffering is a common human experience as opposed to alienation and disconnection (common humanity versus isolation); and (3) practicing mindfulness, which is accepting suffering while maintaining a balanced awareness as opposed to becoming overly identified with it (mindfulness versus over-identification). Increased kindness and decreased self-judgment, feelings of shared humanity and decreased isolation, increased mindfulness and less over-identification with challenging thoughts and feelings are all characteristics of self-compassion, which is a way of relating to oneself in times of suffering^13^.

Compassion, without “self,” can be easily extended to external circumstances that make life difficult to endure, and sorrow arises due to no fault of one’s own. However, self-compassion is equally applicable when our own mistakes, failings, or personal shortcomings are the cause of our pain. Numerous studies indicate that enhanced positive psychological dimensions like life satisfaction and social connectivity are linked to increased levels of self-compassion. Anxiety, sadness, narcissism, self-criticism, and avoidance are all correlated with lower levels of self-compassion^12,14–16^. Taking self-criticism as the focus of the current research, self-judgment as the counter side of self-kindness tends to interchange with the description of self-criticism. It is defined as “Self-judgment is an uncompassionate response; it refers to being harshly self-critical in instances of pain or failure rather than kind and understanding towards oneself^12,13^.” Hence, the inclination to treat ourselves with compassion and understanding as opposed to with harsh criticism or judgment is known as self-kindness. Acknowledging our imperfections and practicing self-compassion entails letting go of our constant self-criticism^17^. Hence, self-criticism, defined as the contrary of self-compassion, is linked to lower quality of life and higher levels of depression, anxiety, and stress^18–20^.

To further investigate the effect of self-criticism, the connection between self-compassion, acceptance, and action, which also highlights psychological flexibility and emotional well-being, should be discussed^21^. Research has shown that self-compassion is significantly correlated with psychological flexibility processes, including mindful acceptance and defusion, and non-judgment refers to the capacity for acceptance and tolerance to make informed decisions or actions by detaching from unhelpful thoughts^22^. In the same research, Gilbert and his team have also mentioned the importance of the competencies of compassionate action in self-compassion. It refers to the competence for successfully shifting one’s focus, logic, and actions toward preventing and lessening suffering. These interventions could be more distant, treating suffering in the present moment, or they could be immediate^22^. This suggests that self-compassion is crucial in enhancing emotional well-being, and it is important to conduct longitudinal research, potentially making it a valuable component of therapies like Acceptance and Commitment Therapy (ACT). From the perspective of ACT, self-kindness is closely associated with self-acceptance, which ACT facilitates a person’s acceptance of sufferings and negative emotions and extends understanding to oneself^21^. It is documented that mindfulness, observing self, defusion, and acceptance are important elements of both self-compassion and ACT, but with ACT, it further facilitates the commitment to values and action^23^.

The ability to accept and to take action regarding unwanted feelings and experiences has been shifted towards a broader concept, psychological flexibility/inflexibility^24^. This concept is then measured using the Acceptance and Action Questionnaire (AAQ;^25^, indicating a significant association with psychopathology, quality of life, and other psychological well-being measurements. The Acceptance and Action Questionnaire II (AAQ-II) is a widely used measure for assessing psychological flexibility, which is a core construct in Acceptance and Commitment Therapy (ACT). Psychological flexibility refers to staying in contact with the present moment and adapting one’s behavior in a way that aligns with personal values, even in the face of complex thoughts and feelings^26^. A meta-analysis was done on AAQ, and revealed significant prediction of various aspects of psychological outcomes, such as depression, anxiety, overall mental well-being, job satisfaction, and other work-related performances^27^. Most importantly, research has demonstrated that the AAQ plays a role in influencing the effects of other coping mechanisms, such as cognitive reappraisal and ACT^28,29^. The AAQ has demonstrated efficacy across various domains, with higher scores consistently correlating with healthier behaviors such as reduced pain, cessation of smoking, improved eating habits, successful weight loss, and better management of both physical and psychological symptoms^24^. Therefore, based on the reviews on the conceptual interconnection between self-criticism, self-compassion, as well as acceptance and action, a model should be done to reveal and investigate the pathway in reducing self-criticism, as if one has equipped with self-compassion and a high level of acceptance and action competence, and enhancing general well-being. Nevertheless, Bond and his team also emphasized the significance of a broader AAQ that can be applied in diverse situations. This is crucial for investigating the theoretical model and the mechanisms that drive therapeutic and behavioral transformation.

## Theoretical framework

Exposure to harmful, scary, or challenging stimuli that surpass an individual’s ability to manage is referred to as stress^30^. According to the Transactional Model of Stress and Coping introduced by Lazarus and Folkman in 1987^30^, stress is viewed not simply as a stimulus or response but as a multifaceted process that includes perception, evaluation, and adaptation strategies. This model emphasizes the dynamic interaction between the individual and their environment, focusing on the cognitive appraisals and coping strategies employed by individuals facing stressors.

Within the pathways of the theory, it suggests that the mediating role of appraisal—the mental process by which events or stimuli are given meaning—influences the severity of a stress reaction. An individual assesses the stressors’ significance (primary appraisal) and its stress-reduction capabilities (secondary appraisal). Primary and secondary assessments are thought to influence an individual’s coping mechanisms.

In this model, self-criticism is identified as a form of cognitive appraisal. When individuals engage in harsh self-evaluation, they interpret their abilities to manage stressors as inadequate. This type of appraisal can significantly heighten the perceived threat or challenge, exacerbating the stress response and affecting both mental and physical health. As such, consistent self-criticism not only acts as a stressor but also intensifies feelings of inadequacy and anxiety, thereby complicating the coping process.

On the other hand, self-compassion is seen as a crucial coping strategy within this framework. Self-compassion involves treating oneself with kindness, recognizing one’s shared humanity, and maintaining a balanced perspective in moments of failure or difficulty. It supports emotion-focused coping by providing emotional regulation tools that help individuals accept and deal with their feelings rather than harshly judging themselves. By fostering a compassionate attitude towards oneself, individuals can mitigate the negative effects of self-criticism and enhance their resilience against stress.

Furthermore, the model suggests that coping strategies can be either problem-focused, involving direct actions to change the stressor, or emotion-focused, involving efforts to regulate the emotional response to the stressor. In the context of this study, both self-compassion and psychological flexibility (measured by the Acceptance and Action Questionnaire, AAQ) are considered tools to cope with emotional experiences.

**Fig. 1:**
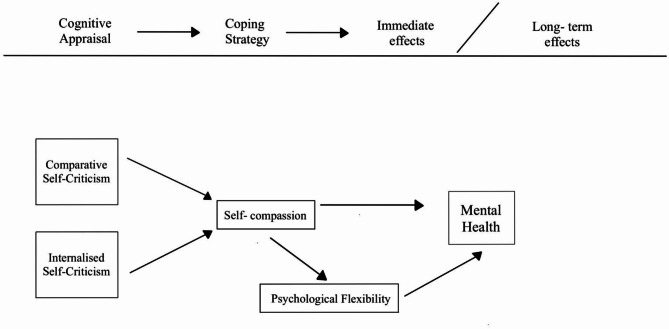
The Transactional Model of Stress and Coping by^31^ adapting to the hypothesized model.

Hence, this study is aimed to examine the longitudinal connections between (1) cognitive appraisal – self-criticism (comparative self-criticism and internalized self-criticism); (2) coping – self-compassion and psychological flexibility (acceptance and action), and (3) outcome – mental health. This approach highlights the significance of individual variances in coping mechanisms and underscores the potential for tailored interventions in fields such as counselling, health psychology, and stress management. Figure 1. Displays the Diagram of the transactional model of stress and coping adapting to the current study hypothesis.

## Methods

### Sampling

This study analyzed data from a longitudinal mental health survey project, which obtained ethical approval at the Chinese University of Hong Kon, and the research has followed the principles of the Declaration of Helsinki for research involving human subjects. All participants provided written informed consent to study participation. Participants were recruited through social media platforms, including Facebook and Instagram, in 2022. The inclusion criteria were as follows: potential participants should: (1) be aged between 18 and 64, (2) provide informed consent and participate voluntarily, (3) be a resident of Hong Kong and currently living in Hong Kong, (4) be able to read and write Chinese, and (5) have Internet access. Participants were excluded if they reported an official diagnosis of a learning or reading disorder, dementia, or cognitive impairments. Eligible participants were invited to complete an online survey consisting of standardized self-report measures at baseline and at 6-month and 12-month follow-up. Part of the data has been reported elsewhere^32^. No incentives were provided for participants.

### Measures

Participants completed questions about demographic backgrounds in addition to the following self-report measures at all time points.

*The single-item measure of self-rated mental health (SRMH).* The single-item measure of SMRH asked, “How would you rate your overall mental health?” (1 = poor, 5 = excellent)^33^. Just like the single-item measure of self-rated health (SRH), single-item measures of SMRH have been widely used in the past decades for public health research^33^. The single-item measure of SRMH had moderate to good test-retest reliability and construct validity in the Chinese context – in particular, the SRMH was highly correlated with a variety of mental health outcomes (e.g., rho = − 0.60 to − 0.66 with depressive symptoms, and rho = 0.69 to 0.82 with self-esteem)^34^.

*The Levels of Self-Criticism Scale (LOSC).* The LOSC is a 22-item reliable and valid measure which assesses the levels of negative self-criticism with two main factors: comparative self-criticism (CSC) and internalized self-criticism (LSC)^5^. The LOSC was translated into Chinese using a collaborative approach^35,36^, with a team of linguist and social workers; its face validity was confirmed by PhD-level psychology and nursing researchers.

*The Self-compassion Scale (SCS)*. The SCS, which has 26 items, is a reliable and valid measure of self-compassion with six factors, including self-kindness, common humanity, mindfulness, self-judgment, isolation, and overidentification^37^. The Chinese version of the SCS was also found to have good internal consistency (α = 0.84) and construct validity, and its six-factor structure was also confirmed^38^.

*The Acceptance and Action Questionnaire II (AAQ-II).* The AAQ-II is a 7-item measure that assesses the levels of psychological flexibility; higher total scores indicate higher levels of psychological inflexibility. The scale shows good internal consistency, test-retest reliability and construct validity^6^. Zhang et al. (2014) reported that the Chinese version of the AAQ-II has adequate reliability, a one-factor structure, and good construct validity^39^.

### Data analysis

The demographic information of the participants and the correlations between variables are recorded using SPSS 28.0. Using Rstudio, the *lavaan* package, the Repeated Measures Panel Analysis Framework in Structural Equation Modelling employs the full-information maximum likelihood estimator (FIML) to investigate the associations between variables and their longitudinal relations simultaneously. This approach enables a better understanding of the dynamic changes of the variables over time. The analysis generates bivariate correlations among all variables in the first wave of the path analysis model. This provides a comprehensive picture of the relationships between the model components, including both direct and indirect effects. The second and third waves of the path analysis model are then analyzed, and the relationships among the model components across three-time points are analyzed. Internal consistency, using Cronbach’s alpha value, and the construct validity, using the Confirmatory Factor Analysis (CFA) of all the measurement models is examined before employing the Repeated Measures Panel Analysis. In this analysis, several indicators are utilized to evaluate the structural model. Firstly, the variation percentage (R^2^) over three-time points is assessed, serving as the stability coefficients for all model components. A variation percentage with fewer fluctuations and a high stability coefficient, defined as 0.70 or above, indicates a consistent structural model^40^. Secondly, model fit is assessed using several metrics: the minimum fit function (χ²), with a chi-square to degrees of freedom ratio ranging from 2 to 5, indicates an acceptable fit^41^. Additionally, the Comparative Fit Index (CFI) and the Tucker–Lewis Index (TLI) are deemed satisfactory when values are 0.90 or above, and between 0.80 and 0.90 refer to as a marginal fit^42^. The Standardized Root Mean Square Residual (SRMR) should be 0.08 or lower^43^, and the Root Mean Square Error of Approximation (RMSEA) should fall between 0.05 and 0.08, with a 90% confidence interval to be considered a good fit model^44^.

## Results

At baseline, a total of 412 participants met all inclusion criteria and provided a valid response. Of these participants, their ages ranged from 18 to 64 (M = 39.73; SD = 12.75). Most of them were female (83.0%) and unmarried (62.6%); 59.6% had an undergraduate degree; 29.1% reported seeing a psychiatrist in the past year. At 6-month follow-up, 232 participants completed the follow-up survey. At 12-month follow-up, 115 participants completed the follow-up survey. For robustness and to maintain the integrity of the analyses, we have included only participants who completed all necessary assessments at the respective time points in the two-wave and three-wave analyses. This was intended to eliminate any issues stemming from missing data. To address the potential selection bias and the reduced statistical power, independent sample t-test, and chi-square analyses showed that participants who completed the follow-up surveys (*n* = 115) and those who only completed the baseline survey (*n* = 297) did not differ in major variables at baseline, including SRMH (*p* = 0.230), LOSC (*p* = 0.055), SCS (*p* = 0.072), female gender (*p* = 0.365), and being married (*p* = 0.803).

However, participants who completed the follow-up surveys (*n* = 115) were more likely to have an undergraduate degree (65.2% vs. 53.2%)(*p* = 0.027), slightly older (t = 2.259, *p* = 0.024), and scored slightly higher on the AAQ-II (t = 2.048, *p* = 0.041).

The outcome measures showed significant correlations with each other (Table 1.)

**Table 1:**
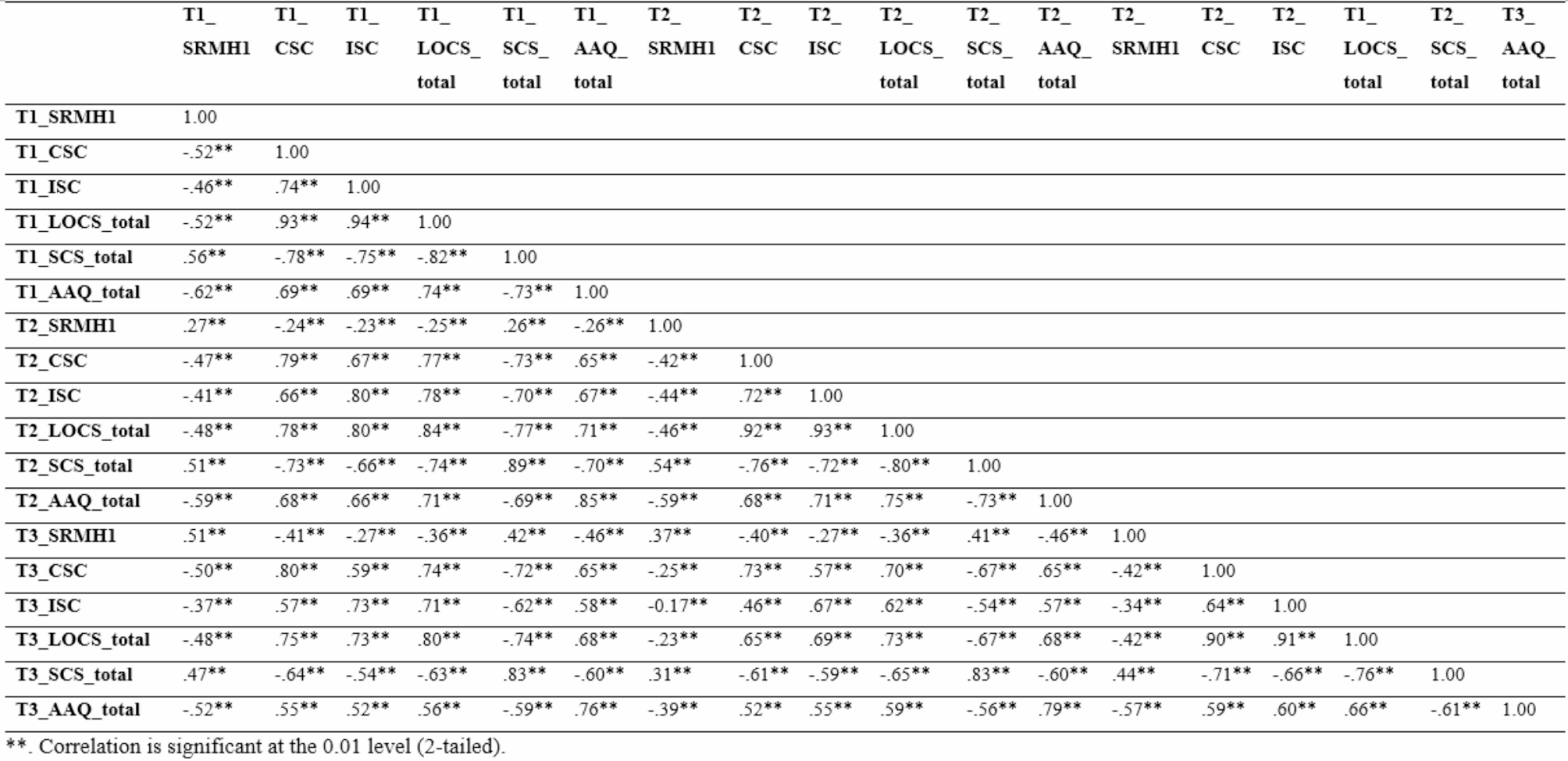
Correlation matrix of indicators.

*p < 0.05, **p < 0.001, T1 = Time 1, T2 = Time 2, T3 = Time 3; CSC = Comparative Self-criticism; ISC = Isolated Self-criticism; SCS = Self-compassion; AAQ = Acceptance and Action Questionnaire (Psychological Flexibility); SRMH = Self-rated Mental Health.

### Confirmatory factor analysis

All outcome measures, SCS, LOSC (two-factor model), and AQA-II, are put into CFA. Most of the measurement models showed a satisfactory fit to the data. However, the LOSC (Level of Self-Criticism) model showed a marginal fit with a CFI of 0.85 and a TLI of 0.82, which are below the threshold of 0.90 but close enough to consider further. It showed marginal goodness of fit but was still acceptable to include in the Repeated Measures Panel Analysis Framework. Please refer to Table 2 for the summary of the goodness of fit index of the measurement models based on the Time 1 data. SRMH shows significant correlations with all the other outcome measures, indicating concurrent validity.

**Table 2:** Summary of the reliability and goodness of fit index of the measurement models.

Model	Cronbach’s alpha	X^2^	df	CFI	TLI	RMSEA	SRMR
SCS	0.94	764.37/284 = 2.69	284	0.92	0.91	0.064 (90%CI = 0.059–0.070)	0.05
LOSC	0.92	904.57/188 = 4.81	188	0.85	0.82	0.095 (90%CI = 0.08–0.10)	0.08
AQA-II	0.94	109.89/14 = 7.84	14	0.96	0.94	0.13(90%CI = 0.11–0.15)	0.03

CFI - Comparative Fit Index; TLI - Tucker–Lewis Index; RMSEA - Root Mean Square Error of Approximation; SRMR - Standardized Root Mean Square Residual.

### The repeated measures panel analysis framework

**Fig. 2:**
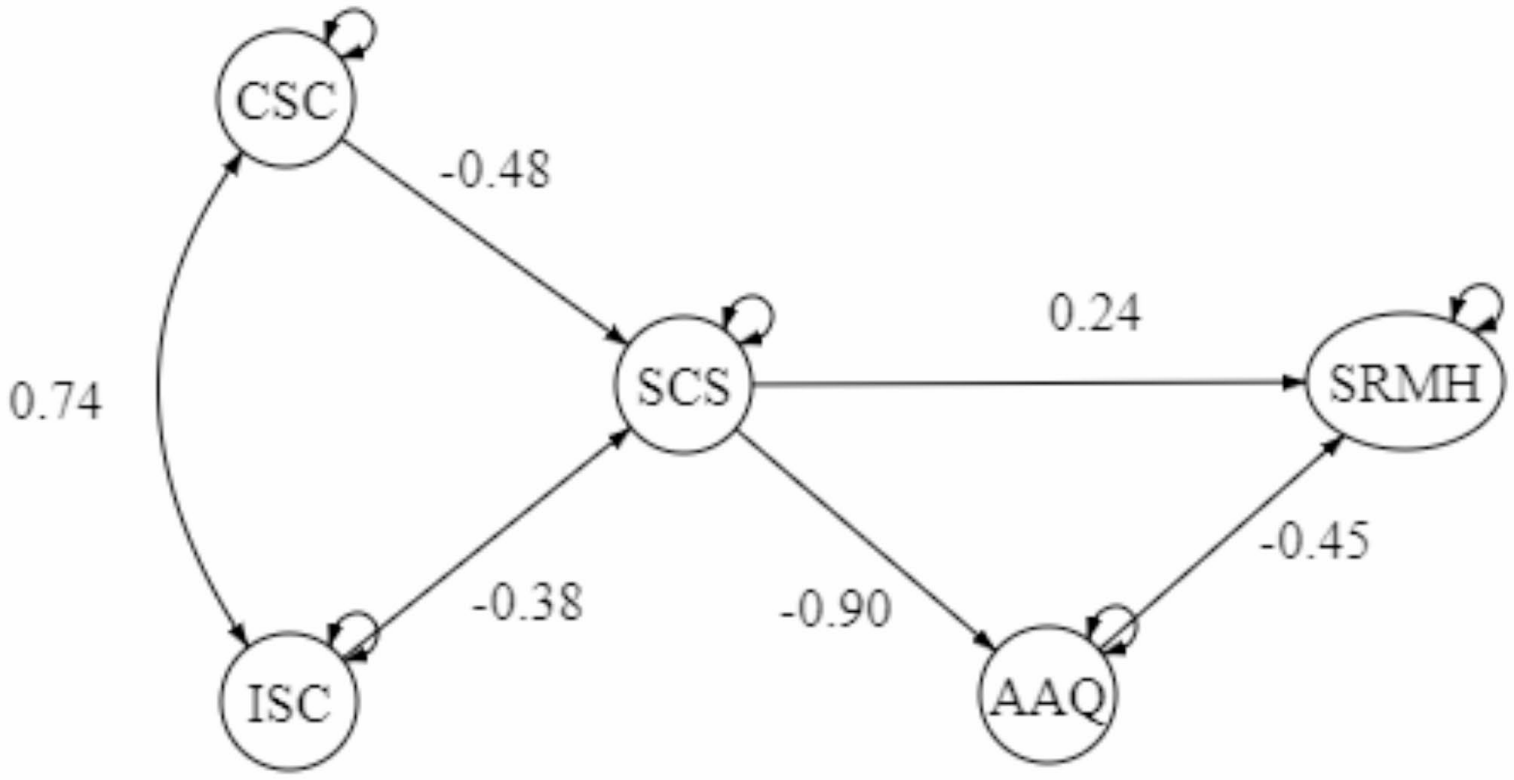
Single-wave model. Path coefficient in β; All paths *p* < 0.05; CSC = Comparative Self-criticism; ISC = Isolated Self-criticism; SCS = Self-compassion; AAQ = Acceptance and Action Questionnaire (Psychological Flexibility); SRMH = Self-rated Mental Health.

**Fig. 3:**
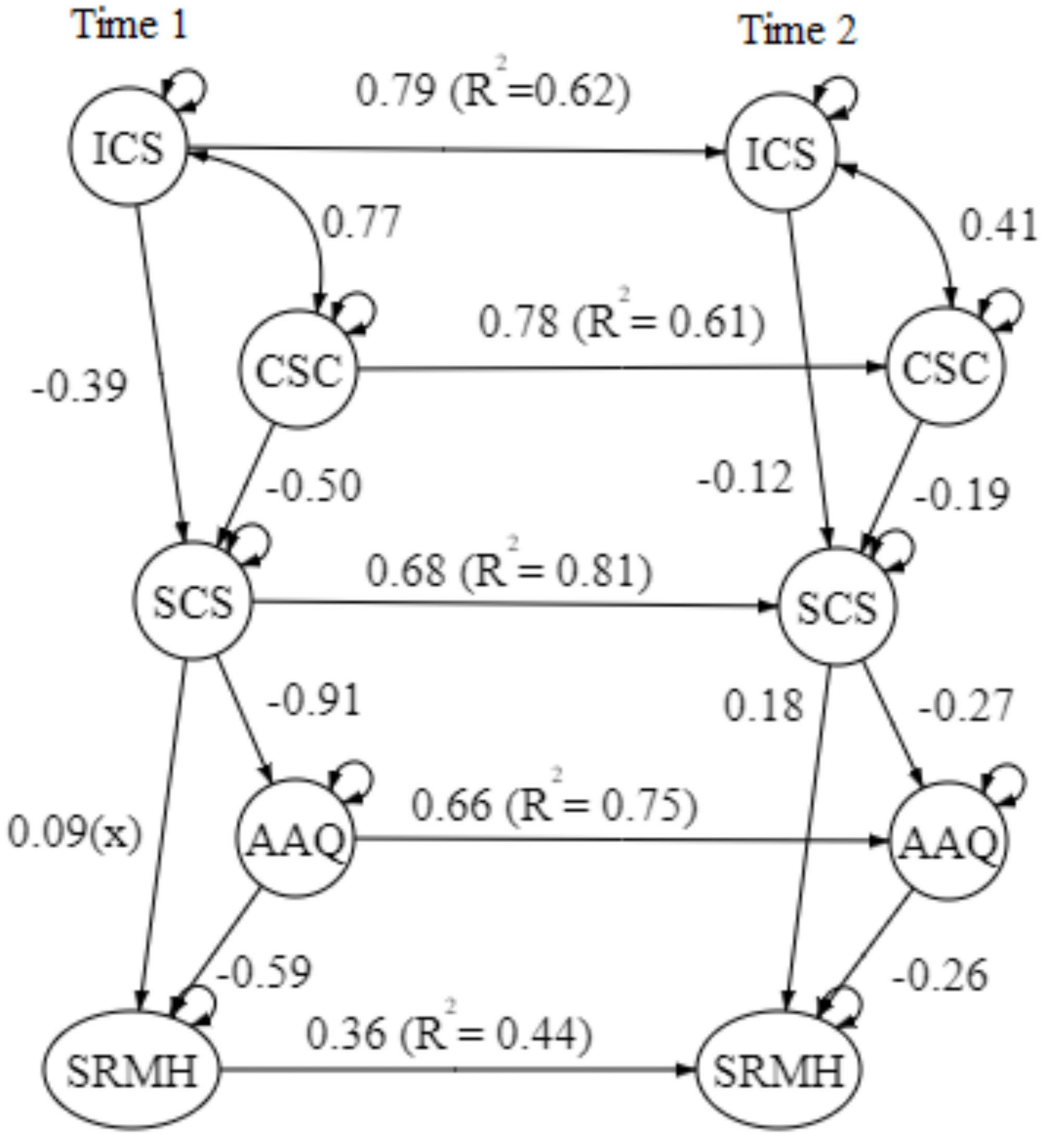
Two-wave model. Path coefficient in β; All paths *p* < 0.05, except with (x); R^2^ = Variation percentage; CSC = Comparative Self-criticism; ISC = Isolated Self-criticism; SCS = Self-compassion; AAQ = Acceptance and Action Questionnaire (Psychological Flexibility); SRMH = Self-rated Mental Health.

**Fig. 4:**
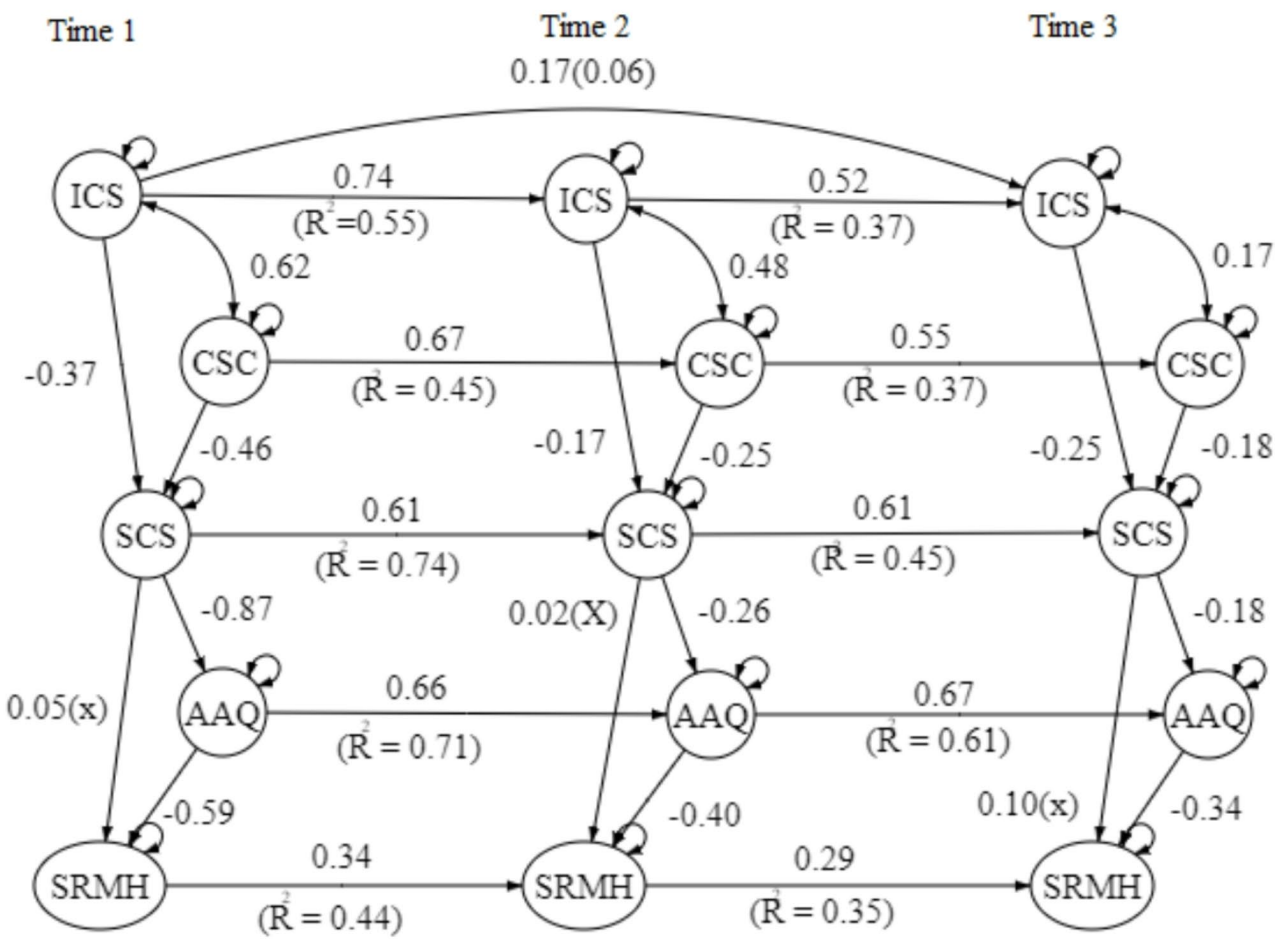
Three-wave model. Path coefficient in β; All paths *p* < 0.05, except with (x); R^2^ = Variation percentage; CSC = Comparative Self-criticism; ISC = Isolated Self-criticism; SCS = Self-compassion; AAQ = Acceptance and Action Questionnaire (Psychological Flexibility); SRMH = Self-rated Mental Health.

The hypothesized model for the first time-point (1st wave) was examined using the Path analysis and resulted in a satisfying good fit model, X^2^ = 5.68/3 = 1.89, CFI = 0.99, TLI = 0.99, RMSEA = 0.047 (90%CI = 0.00-0.05), SRMR = 0.009. Please refer to Fig. 2 for the Path Analysis Model. To examine the longitudinal effect of the model, both 2nd wave and 3rd wave models showed a good fit model, with most of the pathways with R^2^ ranging from 0.6 to 0.8, except having SRMH showed a more fluctuating change among the three-time points, with R^2^ at around 0.35–0.44. The goodness of fit index outcome of the 2nd wave model is, X^2^ = 116.44/27 = 4.31, CFI = 0.96, TLI = 0.93, RMSEA = 0.12 (90%CI = 0.098–0.14), SRMR = 0.067; while the 3rd waves model is, X^2^ = 227.70/74 = 3.08, CFI = 0.91, TLI = 0.87, RMSEA = 0.13 (90%CI = 0.12–0.15, SRMR = 0.18). Please refer to Figs. 3 and 4 for the 2nd and 3rd wave models. In response to the significant attrition observed in the second and third waves of our study, the ‘*semPower*’ package in R is utilized to conduct a post-hoc power analysis for the respective models. These analyses revealed that the statistical power for Wave 2 and Wave 3 models hovered between 50 and 60%, indicating a moderate power level. Importantly, the dropout did not critically undermine the validity of our outcomes.

The repeated measures panel analysis reveals temporal interdependencies among the constructs, indicating that earlier levels of self-criticism predict subsequent levels of psychological flexibility (as measured by the AAQ) and mental health outcomes (assessed through the SRMH). These findings suggest a longitudinal relationship where self-criticism at earlier time points is associated with changes in psychological flexibility and mental health outcomes at later time points. Furthermore, levels of self-compassion have been identified as significant mediators over the three-time points studied. This indicates a potential pathway through which interventions to reduce self-criticism could positively impact both psychological flexibility and mental health over time.

Furthermore, 1000 bootstrap samples were implemented to estimate robust confidence intervals for all model parameters. The results indicated the bootstrap confidence intervals for both direct and covariance paths in the structural equation model. Firstly, there was a positive covariance between ISC and CSC, suggesting a strong relationship between these two subscales. Secondly, there was a significant negative effect between ISC and CSC, suggesting that higher levels of both forms of self-criticism lead to lower levels of self-compassion. Thirdly, there was a large and significant negative effect (est = -14.71, SE = 0.66, *p* < 0.001), indicating a strong inverse relationship between self-compassion and AAQ score. Finally, the negative effects between SRMH and AAQ suggest as the level of mental well-being increases, psychological inflexibility decreases. The confidence interval does not include zero, indicating a significant effect. The two-wave and three-wave models also show similar patterns, thus confirming the models’ pathways and the meditation effect of the variables.

## Discussion

### General observations and key findings

This research aims to investigate the association between self-criticism and mental health and indicate the mediating role of self-compassion and psychological flexibility within this relationship. In the Transactional Model of Stress and Coping context, self-compassion and psychological flexibility are expected to act as the coping strategy to reduce self-criticism and enhance positive well-being. In this study, the model investigation with the good fit model index supports the hypothesized model pathways based on the Transactional Model of Stress and Coping. It confirms the significant mediating role of self-compassion and psychological flexibility in self-criticism and mental health and demonstrates consistency over time. Aligning with the current research, based on the framework of the Transactional Model of Stress and Coping^31^, the influence of self-criticism on stress or even psychosis has been investigated^45^. The same research indicates that self-reassurance, defined as the capacity to concentrate on one’s strengths and exhibit self-compassion during adverse situations, serves as an easing factor against self-criticism and thus seems to protect against psychosis^3,46^.

Additionally, this study indicates that the models’ consistency over time is viewed as evidence of self-compassion, while acceptance and action serve as consistent and stable coping strategies for reducing self-criticism. The outcomes align with the existing literature, stating that individuals who are kinder to themselves are considered as protective from distressing experiences^47,48^. At the same time, self-criticism and fear of self-compassion tend to avoid negative internal experiences more frequently^49,50^. Noteworthy, Moroz & Dunkley (2019)’s study, also a three-wave longitudinal study, supports experiential avoidance mediates the association between self-critical perfectionism and consequent anxiety and depression symptoms.

### The role of self-compassion and psychological flexibility

*“Self-criticism is the process of negative self*-*evaluation.”* High levels of self-criticism are associated with various forms of psychopathology, including depression, anxiety, and poorer therapeutic outcomes^51^. A study highlighted that self-criticism can lead to internal attributions of failure and setbacks, which exacerbates depressive symptoms^52^. These research studies, including a systematic review, indicated the effectiveness of self-compassion intervention in reducing self-criticism. The systematic review and meta-analysis found that self-compassion-related interventions produce a medium reduction in self-criticism compared to control groups^51^. A review on self-compassion programs also mentioned that self-compassion functions as an active component by focusing on processes like self-criticism, which are essential to anxiety and depression^53^. Apart from self-compassion intervention, self-compassion-related therapies like compassion-focused therapy (CFT) and acceptance and commitment therapy were found to significantly improve self-compassion and reduce self-criticism, anxiety, and depressive symptoms^54^. Another meta-analysis specifically focused on CFT found that it effectively reduces self-criticism. This study highlighted that CFT helps individuals develop a more compassionate inner dialogue, reducing self-critical thoughts and behaviors^55^. This evidence supports the effectiveness of self-compassion and compassion-based therapy in reducing self-criticism. Our findings also imply that CFT focusing on improving self-compassion may also help reduce subsequent levels of experiential avoidance, which was measured using the AAQ in the present study.

Nevertheless, psychological flexibility is also considered a coping strategy that is wanted to be evidenced in the current model investigation. Based on the literature review, psychological flexibility is measured using AAQ, while the respective intervention approach is mainly acceptance and commitment therapy^56^. For example, a study found that ACT-based treatments led to meaningful improvements in psychological flexibility, which in turn reduced symptoms of anxiety and depression^57^. Six key processes—acceptance, cognitive defusion, being present, self as context, values, and committed action—are used by ACT to promote psychological flexibility. These procedures support people in committing to behaviours consistent with their values and accepting their thoughts and feelings rather than resisting them^56^. Acceptance and Commitment Therapy (ACT) and Compassion-Focused Therapy (CFT) are evidence-based therapeutic approaches. Also, they both incorporate mindfulness practices and emphasize the importance of accepting rather than avoiding or isolating difficult emotions. However, in contrast to ACT, which emphasizes accepting thoughts and feelings and committing to actions that are in line with personal values even in the face of difficult emotions, CFT focuses on developing compassion and self-soothing skills to counteract the negative effects of self-criticism and shame^58^. This is achieved through mind training and encouraging acceptance exercises^59^. In fact, combining ACT and CFT in an intervention can offer a synergistic approach to dealing with self-criticism, which allows individuals to not only accept their thoughts and emotions but also respond to them with self-compassion^60^. These book chapters have indicated the initial elements of a compassion-focused ACT, showing that this combined approach can provide a more comprehensive toolkit for emotional regulation, facilitating the development of a compassionate and value-driven approach to life and promoting overall well-being. Based on the above, exploring the impact of CFT with ACT or community intervention programs, including self-compassion and acceptance elements on non-clinical and clinical populations, should be done in the future as possible implications.

### Limitations of current mental health measurements

In the model, internalized self-criticism and comparative self-criticism showed a significant correlation; both were negatively related to self-compassion. Self-compassion leads to subsequent mental well-being, directly and indirectly, through psychological flexibility. These pathways showed consistent overtime with R^2^ stabilized at around 0.6–0.8. However, the longitudinal pathways indicating the level of mental health showed inconsistency with R^2^ = 0.35 and 0.42. The fluctuations in R^2^ values might indicate changes due to external influences. This fluctuation may be explained by the usage of the single-item measure of mental health, having the participants respond to “How would you rate your overall mental health?”. The wording of this statement has led the participants to express their current mental health status. This may not accurately represent long-term mental health patterns that are more stable over time. Therefore, responses may be influenced by temporary factors, such as recent events or current stress levels, potentially leading to variability that does not accurately reflect the individual’s typical mental health status. Yet, despite fluctuations in a person’s mental state due to daily activities, the consistent relationship between mental health and coping strategies—such as self-compassion and psychological flexibility—underscores the lasting influence of self-compassion on negative mental well-being.

### Limitations and conclusion

As a longitudinal study analyzed cross-sectionally, the limitation of this study is not being able to examine the causal relationship or implementing effect of self-compassion and psychological flexibility within the relationship between self-criticism and mental health. Despite the three-time point data being an essential contribution to the literature and the framework, the loss of data at the second and third-time points also impacts the statistical results to a certain extent. The significant dropout rate observed in our study appears to have been random, which introduces variability in the sample sizes available at time points two and three. Our analyses revealed that participants who did and did not participate in the follow-up surveys did not differ in major variables at baseline, with a few exceptions. Moreover, the sensitivity analysis using the “semTools” also showed all models comply with the alpha level of 0.05. Despite that, the attrition could potentially introduce biases that affect the validity and generalizability of our findings. Specifically, with fewer participants at later stages, the power to detect meaningful effects and maintain statistical robustness is reduced. Future studies should consider how to reduce attrition rates in longitudinal studies so that the results can be generalized to a broader context. Another limitation is the use of the SRMH. While the SRMH is very useful for large-scale studies due to its brevity, it might not be able to capture mental health constructs as compared to multi-item scales. Nevertheless, we considered the fact that the SRMH was highly correlated with other mental health variables^34^ and that we wanted to alleviate participant burden in completing lengthy surveys, we decided to use the SRMH in this longitudinal study.Hence, a longitudinal study with an intervention will be considered impactful for future studies. In contrast, the current study outcome serves as a theoretical foundation that supports the development of the intervention, evidenced by the potential mediating role or function of self-compassion or compassion-focused therapy and enhancing psychological flexibility through acceptance and commitment therapy.

## Data Availability

The dataset generated and analyzed during the current study is available from the corresponding author (HWF) on reasonable request.
